# Concatenation of Transgenic DNA: Random or Orchestrated?

**DOI:** 10.3390/genes12121969

**Published:** 2021-12-10

**Authors:** Alexander Smirnov, Nariman Battulin

**Affiliations:** 1 Laboratory of Developmental Genetics, Institute of Cytology and Genetics SB RAS, 630090 Novosibirsk, Russia; smirnovskaven@bionet.nsc.ru; 2 Institute of Genetic Technologies, Novosibirsk State University, 630090 Novosibirsk, Russia

**Keywords:** transgenic animals, concatemers, double-strand breaks (DSBs), homologous recombination (HR), non-homologous end-joining (NHEJ), palindromes, repeat-induced gene silencing (RIGS), pronuclear microinjection

## Abstract

Generation of transgenic organisms by pronuclear microinjection has become a routine procedure. However, while the process of DNA integration in the genome is well understood, we still do not know much about the recombination between transgene molecules that happens in the first moments after DNA injection. Most of the time, injected molecules are joined together in head-to-tail tandem repeats—the so-called concatemers. In this review, we focused on the possible concatenation mechanisms and how they could be studied with genetic reporters tracking individual copies in concatemers. We also discuss various features of concatemers, including palindromic junctions and repeat-induced gene silencing (RIGS). Finally, we speculate how cooperation of DNA repair pathways creates a multicopy concatenated insert.

## 1. Historical Overview of the Concatenation Studies

Concatenation is a common term to describe a process of linking monomers together. In nature, DNA concatenation could be used by viruses to amplify genome copy numbers [1,2]. Alternative applications of this principle include mitochondrial DNA replication [3], telomere maintenance [4], and multimerization of human artificial chromosomes [5] or replicative plasmids [6]. We will use the term “concatenation” in the context of transgene delivery, when tandemly arrayed transgene copies co-integrated into a genomic site. This process differs from plasmid multimerization, because after injection of hundreds of molecules their ends are recognized as double-strand breaks (DSBs) in a swift and chaotic fashion, determined by a complex interaction of DNA repair pathways.

Concatenated DNA was found in all model organisms, including mammals (pronuclear microinjection) [7], plants (microprojectile bombardment/T-DNA transformation) [8,9], or nematodes (gonad injection) [10]. Concatenation is a constant companion of transgenesis in cell culture and embryos and was reported for all types of DNA donors, including lentiviral backbones [11], bacterial artificial chromosomes [12,13], transposons [14], Cas9-cleaved minicircles [15], and single-stranded ODNs [16,17]. In this review, we will mainly focus on the mouse pronuclear microinjection as one of the most studied and standardized methods.

**Figure 1:**
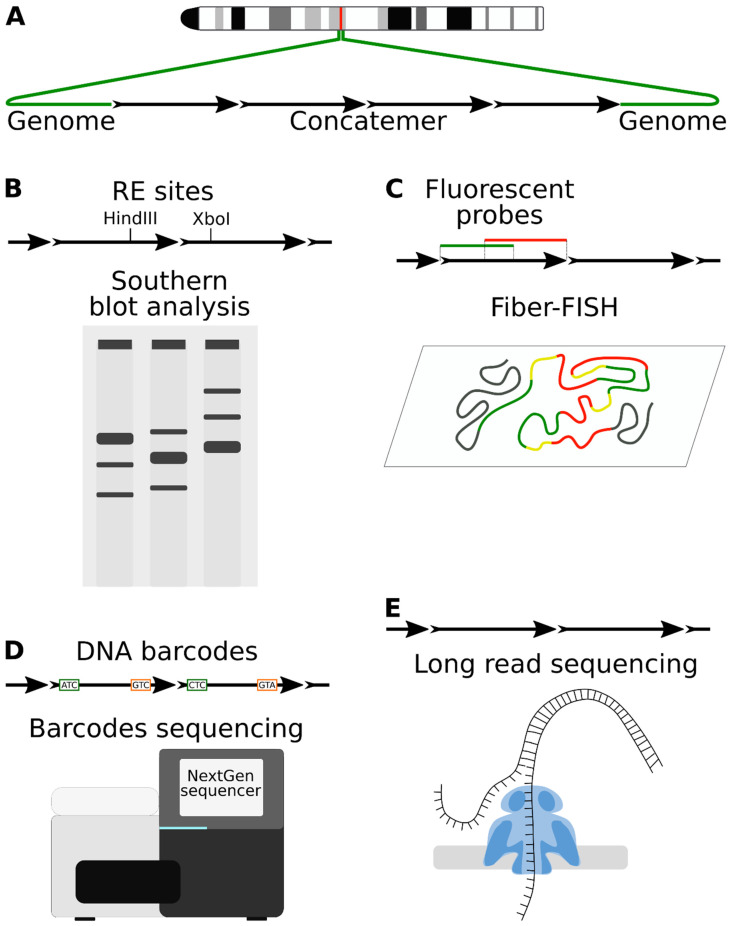
Transgene concatenation and recombination reporters. (**A**) Schematic representation of the concatenated tandem repeat at the chromosomal locus. (**B**) Transgene copies could be labeled with alternative restriction sites to study concatemer by Southern blot. (**C**) FISH analysis on extended DNA fibers demonstrates tandem organization of concatemers. (**D**) Barcoded plasmid library could be used to investigate concatenation. Barcodes from the transgenic copies are amplified with PCR and analyzed by NGS. Connections between barcodes are used to model continuous chains of copies. (**E**) Oxford Nanopore technology, a real-time single-molecule sequencing method, is becoming popular for concatemer analysis.

Microinjection of DNA into the pronucleus of zygote is the main route for obtaining transgenic animals [18]. The effectiveness of this method was first demonstrated almost 40 years ago [19,20]. During microinjection, around 1000 copies of the linear transgene molecules enter the pronucleus. Until the moment of integration, they recombine into tandem repeats (concatemers) containing up to several hundred copies of the transgene and in the overwhelming majority of situations directed in tandem (“head-to-tail”) (Figure 1A). The fact that in most cases, the transgenic DNA is found in the form of multiple repeated monomers (tandems) located at a single locus [21,22] predicts the existence of extrachromosomal concatenation mechanisms. This phenomenon has been extensively studied by many research groups in embryos [23,24,25,26] and injected [27,28] and transfected cells [29,30].

Several explanations for the phenomenon of concatenation have been proposed. One model predicts that a concatemer is built from one or more circular copies through de novo amplification by a rolling circle amplification or other means. This model could be inferred from the high efficiency of concatenation, the notion of occasionally observed identical transgene-transgene junctions [31], and the fact that cells possess mechanisms for gene amplification [5,32,33]. However, the amplification hypothesis was disproved by the studies with concatenation reporters, which demonstrated that copies in the concatemers are unique (Figure 1B–D) (see below). The second hypothetical mechanism of concatenation can be referred to as the “overlapping fragment model” [7,21]. Injection of the supercoiled plasmid molecules can produce concatemers [28]. In theory, occasional breakage of circular molecules will result in a pool of overlapping homologous DNA fragments that would efficiently recombine in the zygote. Finally, the most “down-to-earth” hypothesis postulates that concatemers are formed by homologous recombination (HR) between the linear ends of transgenic molecules with some contribution from circular copies [32,33]. However, this model does not explain why linear ends are preferentially processed by HR factors rather than subjected to random non-homologous end-joining (NHEJ). None of the proposed models of transgene concatenation had been unequivocally confirmed, even decades after their appearance, because analysis of repetitive DNA recombination was difficult with the existing methods.

Understanding the structure of concatemers is important and could be achieved only by labeling copies with individual signatures. There were only a few experiments with such reporters. One notable work from the laboratory of the future Nobel prize winner Mario Capecchi came out almost 40 years ago [27] but is often cited today. The researchers injected cultured cell nuclei with varying numbers and shapes of DNA molecules (linearized and circular). The number of molecules varied from 2 to 1000. To study the concatenation mechanism, they marked the injected molecules with primitive “barcodes” (HSVtk gene in two orientations) [27]. Molecules with alternative HSVtk variants (A/B variants) generated different signals on the Southern blot after restriction (Figure 1B). Analysis of concatemers from cell clones showed that they consisted of two mixed variants of molecules. This implied that concatemers assemble from several linear copies, although the resolution of the method did not allow to reject the amplification hypothesis with confidence. One of the main conclusions of this pioneering work was that the copy number of inserts correlated with the concentration of injected DNA and that the presence of linear DNA ends stimulated concatenation, which also hinted at the assembly of concatemers from individual molecules rather than one amplified circular copy. The follow-up analysis with similar constructs demonstrated that there is a narrow time window, when DNA molecules could recombine, because separating A/B injections by one hour results in a biased ligation of copy variants into uniform repeats rather than interspersed concatemers [28]. 

After some time, this approach was re-explored with a different reporter. In the updated assay, a direct repeat of two LINE-1 elements was injected in the pronuclei [34]. The two copies were identical except the restriction sites, which were used for Southern blot analysis. The amplified construct would have provided a single band, while recombination between copies would lead to a change in the restriction site order in concatemer. Initial Southern blot analysis indicated that injected molecules exchanged sites during concatenation. In addition, thorough analysis of the concatenated inserts was undertaken after plasmid rescue. The authors described several complex recombination patterns, including deletions and duplications inside the tandem LINE1 reporter, indicating the intermolecular recombination [34].

Concatenation was considered as a promising tool for assembling lengthy transgenes from fragments directly in the zygote. Transgenic mouse lines carrying human albumin (hSA), assembled from 7 + 13 + 17 kb fragments, or human adenosine deaminase (ADA) (29 + 39 kb fragments), were created by pronuclear microinjection [35,36]. In the first case, the overlapping regions of the fragments were about 2–3 kb, and in the second, 19.5 kb. Southern blot analysis confirmed that correct copies of the gene were present in most of the transgenic offspring, although incomplete copies or rearrangements were also encountered [35]. Of note, there was little concatenation between identical fragments. In a similar work, the human lipoprotein receptor (VLDLR) gene was successfully assembled from two fragments of 40 and 35 kb, with an overlap of 8 kb [37]. The authors used FISH analysis on extended DNA fibers (Fiber-FISH) to visualize a stretch of head-to-tail tandems in the genome of transgenic animals (Figure 1C). Later, this concatenation approach was abandoned, because microinjection of BACs into the pronucleus proved to be effective [38,39].

We have suggested improving the concatenation reporter by combining DNA barcoding and next-generation (NGS) sequencing (Figure 1D). We created a plasmid library with thousands of unique barcode pairs separated by a restriction site. Note that in this assay, barcodes are located close to the exposed ends of the construct, rather than inside the backbone. Linearized copies with barcoded ends are injected into the pronucleus, which causes concatenation and ligation of barcoded ends together. Barcodes from transgenic animals are amplified with PCR and identified by NGS sequencing (Figure 1D), providing plenty of data for analysis. This reporter can discover copy amplification cases. Connections between the barcodes, observed in the NGS data, are used to build a chain of molecules, and understand the order of copies in concatemer. Unfortunately, only direct head-to-tail repeats could be detected because PCR amplification of inverted repeats is ineffective [40].

Recently, single-molecule sequencing technologies, e.g., Oxford Nanopore technology (Figure 1E), made a revolutionary step forward for genome analysis (see the corresponding chapter of this review). These methods allow study of the complex structural variations and palindromes, because PCR amplification is not required in sample preparation. Ironically, the story of concatenation began way back before the invention of PCR and made a comeback when PCR went out of fashion.

## 2. Information from the Internal Junctions

**Figure 2:**
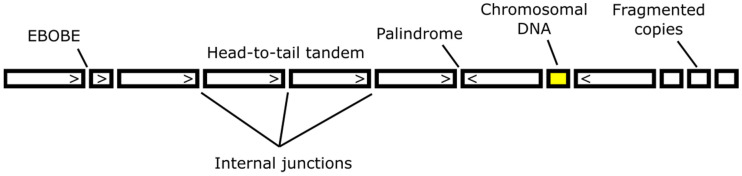
Concatemer elements that could be present at the integration site (see explanations in the text).

Sequencing internal junctions between transgene copies (Figure 2) was a primal source of data about concatemer structure before invention of the methods that allow the study of concatenated repeats, such as long-range sequencing or DNA barcoding. In the nucleus, linear transgene ends are recognized as DSBs by DNA repair machinery. Non-homologous end-joining is a primary repair pathway in embryos and embryonic stem (ES) cells, while microhomology-mediated end-joining MMEJ serves as a backup plan and uses small homologies (5–25) revealed from DNA resection [38,41]. MMEJ is responsible for 10–20% of DSB repair and competes with HR at MRN-resected ends [39]. Experiments with genetic reporters in mouse ES cells show that in the absence of classical NHEJ (Ku80−/−, LigIV−/−), 93% of breaks are repaired with microhomologies (>2 nucleotides). Normally, this value is 2–3 times less, depending on the specific locus [42].

The injection of embryos with molecules having different restriction digested ends (5′, 3′, blunt) did not reveal any bias in end repair [32,43]. Most of the time, internal junctions from the injected embryos had only small deletions: clippings of the transgene ends prior to ligation [33,44]. In rare cases, transgenes are re-ligated by sticky ends formed from restriction endonuclease digestion [45]. Thus, most of the internal junctions in concatemers represent NHEJ-ligation products.

The incorporation of concatemers into the genome also occurs due to NHEJ/MMEJ activity. Inactivation of the two pathways through inhibition of Ku70/Ku80/ Lig4 (NHEJ) and polymerase θ (TMEJ, the main MMEJ pathway) reduces the frequency of random insertion of transgenes by five orders of magnitude [46]. The MMEJ signatures are frequently seen when analyzing the nucleotide sequences of the transgenic-genomic boundaries in cells and embryos [47,48,49].

In some cases, additional DNA co-integrates with concatemers. The fragments include chromosomal and mitochondrial DNA, mobile elements, telomere repeats, and bacterial DNA, and are typically found at the transgene-genome junctions but could also localize inside the concatemer [13,50,51,52,53]. 

The hierarchy of the fragment assembly during concatenation that can be devised from the NHEJ/MMEJ signatures at the junctions is an interesting topic for speculation. The frequency of microhomology-mediated joining at the transgene-genome borders seems to be higher than at the internal junctions. This fact might indicate that integration in the genome occurs at the final stages of concatemer formation when most of the ends are resected. Systematic analysis of the contiguous concatemer inserts with single-molecule sequencing will be required to clarify this issue and offer a road map of concatenation.

## 3. Palindromes

Palindromic sequences are broadly represented in the vertebrate genomes [54] and are associated with repetitive sequences, such as short microsatellite repeats [55], mobile elements [56], de novo structural variations in cancer cells [57,58], and sex chromosomes [59,60]. Some prominent palindromes have been extensively studied. For example, in the human genome, palindromic AT-rich repeat (PATRR) is responsible for the frequent reciprocal translocation of regions at chromosome 11 и 22 during spermatogenesis [61], which causes Emanuel syndrome [62].

The presence of palindromes in the genome provokes rearrangements, but there is no consensus on the mechanism of their occurrence. According to the most popular model, replication and transcription of genome regions induce the appearance of single-stranded DNA regions, which leads to the formation of special cruciform structures and hairpins [63,64]. Interestingly, post-mitotic neurons that halted replication can destroy palindromes [55], and introducing replication origin close to the palindrome structure does not increase the frequency of rearrangements [65]. It is also known that the DSB adjacent to the palindromic construct initiates resection, leading to the emergence of single-stranded hairpin-forming regions in DNA [66]. The existence of cruciforms and hairpins in palindromes has been shown experimentally [61,65]. These structures are recognized and bound by many DNA damage response factors [67,68] and cell endonucleases, which process these topologically constrained regions into DSBs. Possible candidates include endonucleases Mre11 [69,70], GEN1 and Artemis [71], ERCC4 (Xpf) [72], or MutL complex [73]. 

Recombination of palindromic junctions is studied using special reporter constructs [56]. The authors obtained insertions in the yeast genome with two types of palindromic fusions: with a large (1000 bp) or short (12 bp) spacer. A spacer is a unique piece of DNA that separates two inverted repeats (palindrome). The palindromic regions themselves were about 2000 bp. This and other experiments demonstrate that even short spacers are capable of stabilizing palindromes, possibly due to prevention of the denaturation at the central point between two inverted repeats [56,74]. 

In another work, an episomal reporter construct was used, in which the GFP gene was interrupted by the insertion of a palindromic region (40 + 40 bp). Experiments were carried out in several types of cells (HeLa, HEK293T, and COS-7) [65]. Positive recombination events (deletions in the palindrome) were detected as a result of the restoration of the GFP gene using a donor plasmid (full-length GFP gene without a start codon). Meganuclease I-SceI and a similar reporter plasmid were used as controls, where an I-SceI site was inserted instead of the palindromic region. It was found that the presence of a palindromic region in the plasmid provokes DSB with a frequency comparable to targeted digestion with nuclease (1–2% of cells). At the same time, the insertion of a small 35bp spacer between inverted copies notably reduced the frequency of breaks, down to the background level [65]. A similar study with palindromic Alu repeats showed similar results and estimated the size of the spacer that blocks rearrangements (52 bp) [63].

So, how can this knowledge translate to concatenation studies?

**Figure 3:**
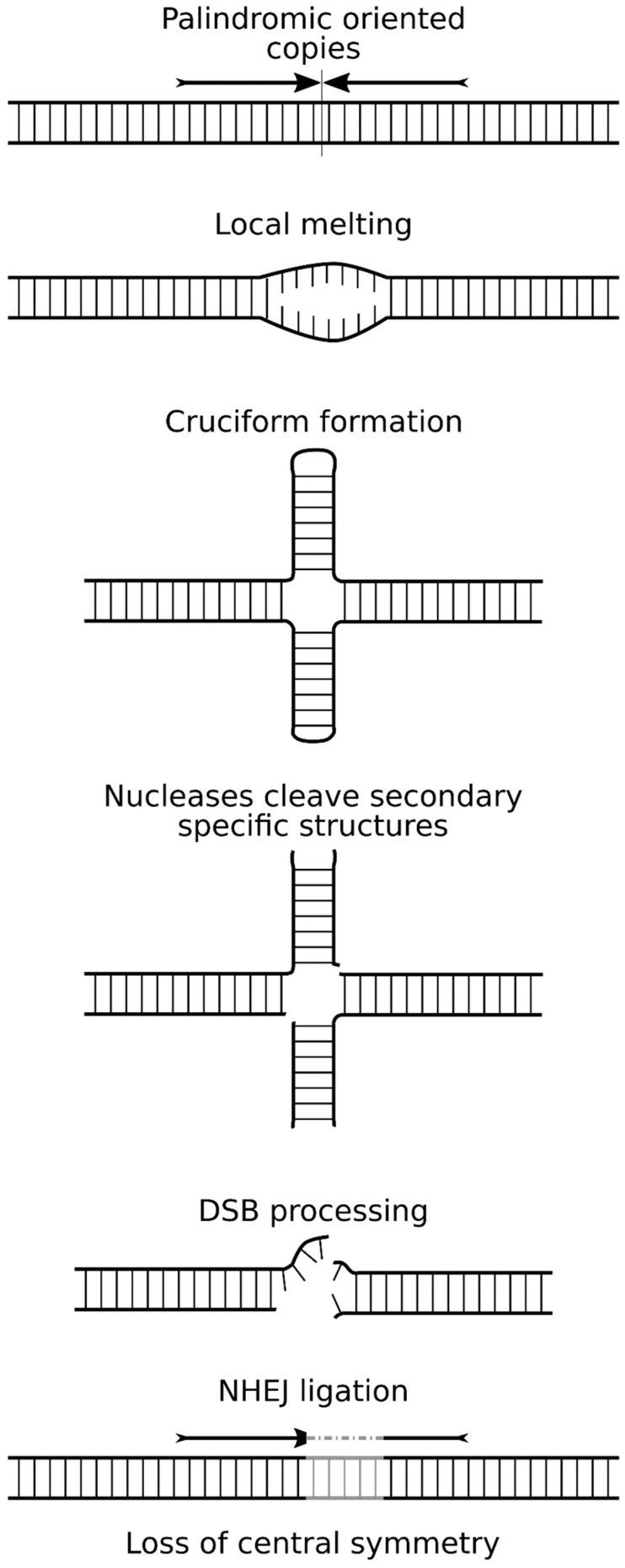
Cells stabilize palindromic junctions by introducing asymmetrical deletions. The general scheme of the appearance of cruciforms and their transformation into DSB. The reasons for denaturation can be different: torsion stress, replication, transcription, close DSB.

Concatemers frequently contain palindromic segments (that is, “head-to-head” or “tail-to-tail” inverted repeats) as a consequence of random transgene ligation [42,53,56]. To understand the process of concatenation, it is important to study palindromic variants of junctions between copies, and their frequency and stability in the animal genome. Concatemers are stably inherited by the descendants of the transgenic founder animal, but the fate of palindromic junctions between copies has hardly been studied in the generations. Long-term observation of palindromic fusion in a transgenic mouse strain has helped to better assess the stability of this structure. In the laboratory of Maria Jasin, experiments were carried out to create mice with a reporter construct from two consecutive copies of the LacZ gene [75]. One of the transgenic lines harbored a palindromic insert of two transgenes equal to 15.4 kb in length. This mouse line represented a rare case of a perfect palindromic junction with no deletions inside the internal junction [75,76,77]. The authors studied the inheritance of the transgene and documented the high frequency of small deletions and more complex recombination products at the junction point in the palindrome in about 35% of the offspring [75,76]. An immortalized cell culture was obtained from mice of this strain [77]. Long-term cultivation of cells with a palindromic structure showed that the average frequency of rearrangements within the palindrome was 5.5 × 10^−3^, which is approximately 0.5% of cells per passage. Most of the rearrangements were small deletions in the palindromic junction, which broke the central symmetry (of the 118 rearrangements analyzed, 93% were deletions). The destruction of the symmetry increased the stability of the palindrome in the subclone by a factor of 25 [77] (Figure 3). In general, the frequency and patterns of rearrangements within the palindrome in mice and cells were very similar.

Unfortunately, palindrome studies in transgenic embryos or mouse lines are rare, because common molecular methods based on PCR are not suitable for detection and sequencing of inverted repeats. This is probably due to the formation of a hairpin in the PCR product, which prevents amplification ([78]; our unpublished data). Single-molecule sequencing methods do not suffer from these limitations and could be used to investigate complex transgene junctions [40,52]. We used the PacBio approach to analyze concatemer with dozens of palindromes. Most of the observed inverted junctions (head-to-head or tail-to-tail) carried asymmetric deletions, which are believed to stabilize the palindrome [40]. An alternative stabilization mechanism (spacer inclusion) was uncommon, probably because the chance of incorporating a DNA fragment between two inverted copies is not high during concatenation.

## 4. Molecular Mechanisms of Concatenation

To understand the molecular mechanisms resulting in concatenation, we should first establish if there really is a preference in copy orientation. In the case of random copy ligation by NHEJ, a distribution of 25%: 50%: 25% can be expected for the three possible types of orientations (50% corresponds to a head-to-tail orientation). Numerous descriptions of concatemers in pronuclear microinjection provide compelling evidence that head-to-tail orientation has an observed frequency over 90%. Importantly, the calculations were made by different methods: Southern blotting [23,24,41,48,79], whole-genome sequencing [49], Oxford Nanopore technology (ONT) [52], and PacBio [40]. There are also cases of very long concatemers (~267 copies) with exclusively tandem orientation of transgenes [80]. Although in some reports observed transgenes are ligated randomly [81], it is safe to assume that head-to-tail concatenation is a characteristic feature of pronuclear microinjection and there must be a mechanism to organize the direction of the copies.

**Figure 4:**
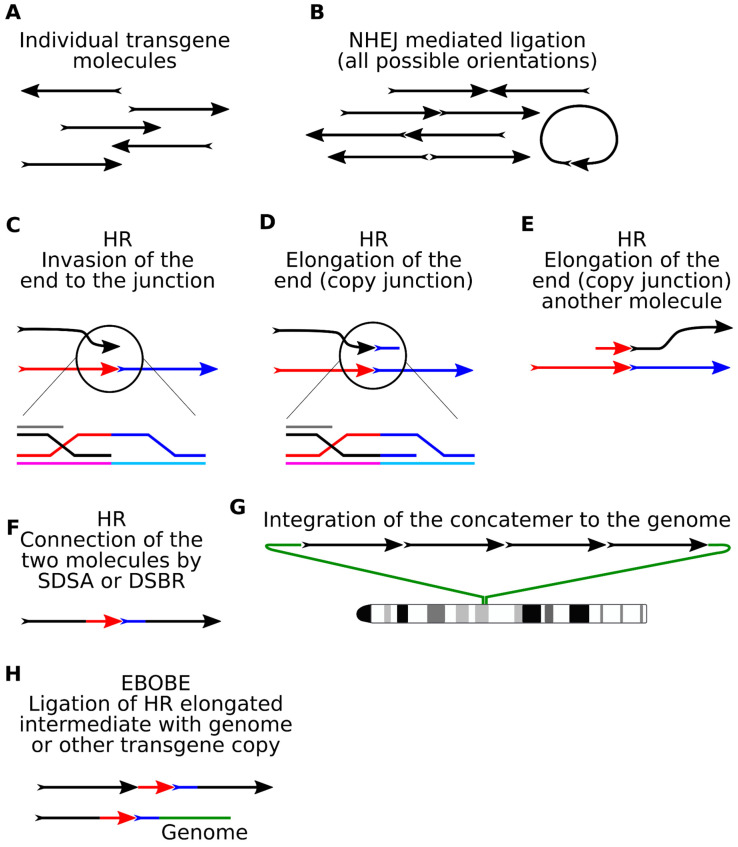
DNA repair pathways that contribute to transgene concatenation. (**A**) Injected transgene molecules. (**B**) Initial legation of copies is carried out by NHEJ, creating templates for HR. (**C**) Resected ends invade donor templates and (**D**,**E**) copy a portion of the junction (blue). (**F**) Re-annealing of the complementary synthesized ends leads to connection of the copies into tandem repeats. (**G**) Integration of the concatemer into the genome is facilitated by NHEJ or MMEJ after concatenation. (**H**) EBOBE pattern—a product of DNA end synthesis that is incorporated into the concatemer after HR termination.

Two main HR mechanisms, synthesis-dependent strand annealing (SDSA) and double-strand break repair (DSBR), participate in the assembly of transgenes into the concatemer tandem (Figure 4C–F). The joint step for both pathways involves initial 3′-resection of double-stranded ends of linear molecules and a subsequent search for a homologous region for invasion [82]. Apparently, this would be the junction site between the two copies. In the SDSA scenario, extension of the end is carried out to achieve overlapping homologies that are patched together in a head-to-tail order. For DSBR, capture of the second resected end is required, then a double Holliday junction (dHJ) is formed, which can be resolved in two ways (with or without crossing over). Cells typically strive to prevent crossovers with the Sgs1 (BLM) helicase activity, which eliminates dHJ by active branch migration and dissolution [79,83]. 

The DNA ends participating in HR are very active, and some molecules could be copied three to five times before finally being integrated into the concatemer [40]. The data from both LINE1 and barcoded reporters show that most of the copies participate in recombination (50% and 80%, respectively) [34,40]. The addition of biotin to the transgene ends blocked concatenation [84,85], demonstrating that linear ends are indeed initiators of recombination. 

Another indication of end recombination was discovered accidentally in the barcoded concatemers and was named Elongation Beyond Original Broken End (EBOBE) [40]. The EBOBE pattern represents intermediate DNA synthesis metabolite, an elongation of the initial resected transgene end that copied the junction up to the barcode (Figure 4H). This heterologous region at the 3′-end prevents the copy from returning for a new round of synthesis, so it joins the concatemer via NHEJ or MMEJ. Such mechanisms are known for some other HR metabolites (non-canonical HR termination) [86] in combination with the activity of theta-mediated end-joining (TMEJ) polymerase θ [87].

Retrospective analysis of the published cases provides examples for the EBOBE pattern. For instance, several reports described a single copy of transgene that was flanked by a truncated copy with almost no clipping at the internal junctions [35,51,88]. These cases have similar traits: minor clipping inside the transgene-transgene junction and a short elongation fragment nearby. 

Unfortunately, it is still impossible to exclude the possibility that two copies were ligated independently. We have revisited this problem with our barcoding approach [40]. In this case, the barcode serves as an EBOBE “trap” that helps to detect these cases. Using this logic, we annotated four cases of EBOBE in long PacBio reads (around 56 copies in total) and three cases, accidentally caught at the transgene-genome junctions in other embryos. 

Some but not all EBOBE fragments had microhomology and were probably processed by MMEJ as the single-stranded HR. 

Similar recombination patterns could be found in the cell culture experiments with targeted DNA integration. It is a common knowledge that homologous arms of the donor vector participate in SDSA-directed integration [89]. Sometimes, the donor cannot be processed completely, and DNA synthesis is halted inside the construct, leaving truncated insertion. In the case of transgene concatenation, HR is initiated not between the genome and the homologous arms, but between the pool of identical transgene copies, and the truncated intermediates are preserved inside the concatemer.

The contribution of other HR repair mechanisms, such as single-strand annealing (SSA), microhomology-mediated break-induced replication (MM-BIR), and multi-invasion recombination (MIR), is unknown. We did not find a significant impact of these pathways on concatenation in our assay. MM-BIR occurs upon invasion of non-homologous regions of the genome due to microhomology at the 3′end of the molecule. MM-BIR would lead to copying of an extended concatemer region (from several thousand to many hundreds of thousand base pairs), which would lead to duplication of a large region [90]. MIR is a novel potentially mutagenic DSB repair pathway [91]. Recent work by Wolf-Dietrich Heyer’s group has demonstrated that resected 3′-filaments can use internal single-stranded regions far from the 3′-end of the filament to search for homology. This leads to the fact that one filament can invade two independent regions of the genome and combine them into dHJ [92], which, when resolving dHJ with crossing over, leads to translocations of chromosomal fragments. Most likely, these mutagenic pathways (SSA, MM-BIR, MIR) are suppressed during concatenation, because their initiation requires extended end resection (>1 kb) [90,91]. In conditions where homologous sites for invasion are in excess, SDSA/DSBR should utilize normal resected ends <1 kb [93] and effectively compete with the aforementioned pathways. In theory, the multi-invasion frequency and template switching during HR in concatenation could be studied with a uniformly barcoded vector library. Instead of barcoding transgene ends, it would require the insertion of multiple barcodes or SNPs every ~1000 bp of the backbone to analyze gene conversion between copies. 

In this chapter, we discussed how high HR activity in the embryo organizes molecules into tandem repeats. Curiously, there is not much evidence about transgene copy orientation in cell cultures. In theory, copy arrangements there would be more random due to NHEJ/MMEJ domination through the cell cycle [88], although non-random head-to-tail concatemers [85] and end recombination (our unpublished data) suggest that concatenated ends could be processes by HR in cells. In addition, transgenic cell clones are obtained by lipofection, electroporation, or viral delivery [94] in the asynchronous cell cultures. It would be interesting to compare concatemers in embryos/cultured cells obtained by the same delivery method (microinjection).

## 5. Repeat-Induced Gene Silencing

Repeat-induced gene silencing (RIGS) leads to expression silencing in high-copy transgene insertions and is established gradually with time in cell passages, embryo development, or in later generations. RIGS exists across all multicellular organisms, including Drosophila [95], fungi [96], plants [97], and mammals [98,99]. In animal biotechnology, it is a general rule to avoid multicopy founders during initial transgene screening in fear of RIGS, but the molecular mechanisms of this process remain unknown in mammals. 

The most perplexing feature of RIGS is that, unlike in gene position effect variegation that is determined by the surrounding regulatory elements [100,101], RIGS occurs regardless of the integration site or specific transgene sequences and can onset later in development [102]. To make things even more complicated, sometimes multicopy concatemers evade RIGS (different constructs show different results), and a positive correlation between the copy number and expression is not rare [12,103].

Another observed peculiarity that could be attributed to RIGS is “expression plasticity”, the notion that the expression of transgenes at a single locus negatively affects each other [104] and that consequent integration of two highly expressed genes can decrease their expression [105]. It was also shown that reactivation of transgene expression by antibiotic selection can activate neighbor transgenes in cis [106].

It is possible to directly interfere with RIGS by Cre-mediated reduction of the transgene copy number at the site (from 8 to 1–2 copies), which led to an increase in the expression [107] in mice, but other experiments using the same Cre-based approach did not find a positive correlation between the expression and copy number reduction at another locus [108]. The fact that even a single copy integration can be silenced with passages adds more complexity to the picture [109]. Strikingly, the expression of the constructs inside safe-harbor loci, such as human AAVS1 or mouse Rosa26, could be silenced in some cases as well [110,111,112].

How can RIGS be explained? In plants, small RNAs generated from the expressed transgenes guide homologous mRNA cleavage for post-transcriptional gene silencing (PTGS) [113]. In addition, plants can silence transgenes through RNA-directed DNA methylation (RdDM), which acts at transcriptional level (TGS) [114]. In Drosophila, a similar process is guided by Piwi-interacting RNAs [115]. Studies in the mouse cells suggested the possibility of RNAi gene silencing associated with convergent transcription dsRNA of palindromic copies, which are common in the concatemers [115]. However, later experiments with a huge 1000 copy tandem reporter [116] unequivocally showed through Dicer knock-down that the RNAi pathway does not establish silencing [117]. 

Alternatively, there is a possibility that RIGS is provoked by the structural changes in the local DNA. Some observations suggest that RIGS could be caused by direct DNA-DNA pairing at repeats and that such recombination-independent recognition of DNA homology could be common in different organisms [118]. 

The correlation of RIGS and repressive epigenetic chromatin marks is well established. DNA methylation is an extensively characterized epigenetic modification (Jones, 2012). The levels of methylation of the CpG residues in the promoter of repetitive sequences generally correlate with the copy number. High levels of methylations are associated with lower gene expression [107,119,120,121,122,123] and silenced transgenes are less sensitive to DNase I and carry repressive chromatin marks [97,124]. Experiments with large silenced repeats have proven that HP1/p150/CAF-1 establish condensed heterochromatin at the multicopy transgenes. This effect was observed in several cell lines [125,126]. 

The most extensive study of the tandemly amplified plasmids, which have similarities to transgene concatemers, originates in the laboratory of Noriaki Shimizu. The group has investigated the causes of RIGS in the IR/MAR plasmid amplification system that lead to extrachromosomal or chromosomal multimerization. Notably, they discovered that inhibition or knock-out of a broad spectrum of histone deacetylases (HDAC1-2, SIRT1) alleviates the RIGS phenomenon [127]. Interestingly, the HDAC inhibition effect only had an impact during initial multimerization of the IR/MAR plasmids. 

It should be noted that this system is not identical to the transgene concatenation during microinjection, because IR/MAR plasmids carry Dhfr selection cassette and Ori sequences that facilitate an alternative multimerization mechanism. Nevertheless, findings from the IR/MAR assay will be relevant to the study of RIGS in concatemers. 

Cell culture experiments aiming at high gene expression employ transposable elements to achieve stable single-copy integrations or induce high-copy amplification in combination with measures to avoid RIGS. These measures may include chromatin relaxation by inhibitors (trichostatin A, butyrate [119]; 5-azacytidine (5-AzaC) [128] or attaching functional elements to the transgene (BAC genomic regions, matrix attachment regions (MARs)) [12,109,129,130].

Obviously, vertebrate RIGS is not a simple phenomenon, and is likely established at multiple levels, including transcription, DNA methylation, and heterochromatinization. Other probable molecular mechanisms include a deficit of transcription factors, epigenetic silencing in meiosis, DNA structural folding, nuclear lamina retention [131], and transcription-induced silencing.

**Figure 5:**
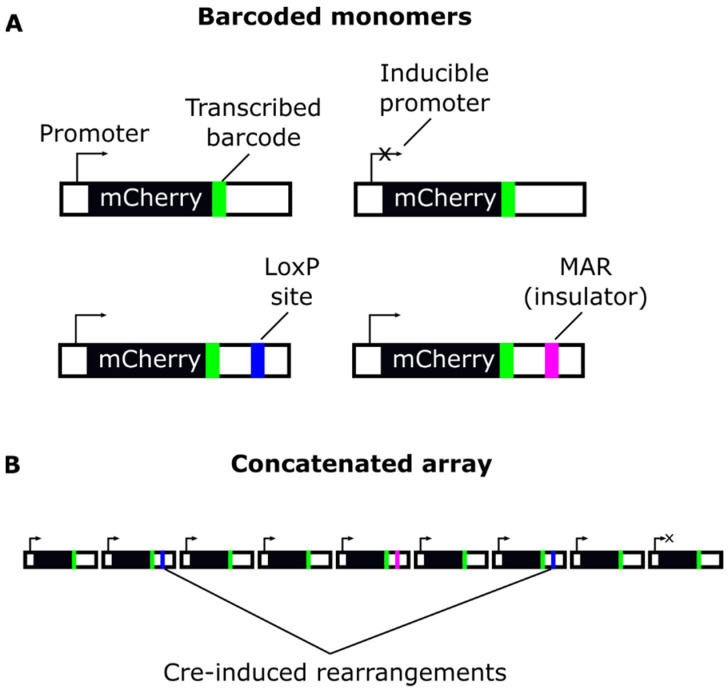
Barcoded reporter for concatenation of expressed monomers to study RIGS mechanisms. (**A**) Types of barcoded monomers. (**B**) Transfection of monomers in specific proportions (e.g., 10:1:1:1, where 10 is the standard monomer) could be used to create unique concatenated arrays for RIGS analysis by RNAseq and other methods. Transfection of the cells with Cre-recombinase would change the order and number of copies.

We intend to employ an expressed barcoded reporter that would be instrumental in studying several aspects of RIGS in cultured cells and transgenic embryos (Figure 5). The reporter is based on a randomly integrated concatemer, consisting of several types of barcoded monomers. LoxP monomers will be utilized to change the order and number of copies [107,108]. Inducible monomers that carry Tet-On promoters will be used to study how reactivation of expression affects RIGS in cis. The inclusion of insulated monomers will show how MAR-elements [103,132] behave in a silenced concatemer. Since the order of copies in the repeat could have an effect on expression [133], it would be interesting to examine if the flanking copies avoid silencing. During concatenation, transgene copies are joined randomly, and the distribution of monomer variants in multicopy integrants will depend on the proportion of monomers in the delivered DNA (Figure 5). Information about individual barcode expression from the RNA seq data could be combined with other modern molecular techniques, such as nanopore methylation sequencing [134], ChIP-seq analysis of transcription factors and chromatin proteins, and dynamic analysis of expression with SLAMseq [135] after HDAC/HP1/DNA methyltransferases (DNMTs) inhibition. This will help to better understand molecular drivers of RIGS and the timeline of events that leads to expression silencing.

## 6. Novel Methods for Studying Transgene Concatenation

Careful readers should have grasped the idea that concatemers are not mere “repeats” of transgene monomers, but they have a complex internal composition scaling with the copy number. Until recently, cataloguing these genetic patterns was impossible because the repetitive nature of concatemers obstructed sequence assembly by next-generation sequencing (NGS). Fortunately, a plethora of novel genomic methods for transgene DNA analysis appeared at the end of the last decade. One of them, targeted locus amplification (TLA), based on proximity DNA ligation is specifically designed to map transgenes [136,137,138]. In this review, we will not focus on it, because TLA mostly provides information about the integration site and the surrounding genomic locus. Undoubtedly, this method will be important for systematic analysis of the thousands of archived transgenic mouse lines [52] to chart integration site preference and analyze collateral genomic damage, including the pervasive presence of the flanking chromosomal duplications that could be found in random and targeted integration approaches [80,129,130,139,140].

Single-molecule sequencing (SMS) technologies will have a huge impact on genomic research, particularly for the study of transgene localization and the internal concatemer structure. Two distinctive approaches are currently being developed: Oxford Nanopore Technology (ONT) and Pacific Bioscience Single-molecule Real-time sequencing (PacBio) [141] (Figure 1D). In ONT, protein pores are integrated into a polymer membrane and an ionic current is passed through the nanopore by setting a voltage across the membrane. A helicase molecule is attached to DNA (or RNA) fragments during sample preparation, which allows molecules to translocate through the pore, generating a change in the current. The signal is deciphered into corresponding nucleotides or their modifications in real time. PacBio uses a different method based on fluorescence detection in the special flow cell picolitre-sized wells. A single polymerase molecule is localized. When a circularized DNA molecule is added to the well, polymerase binds a single-stranded region in the adapter. During complementary strand synthesis with labeled nucleotides, the fluorescence signals are captured through the transparent bottoms of the cells in real time.

Both methods offer great utility for concatemer analysis, as they are suitable for palindrome detection and generate long reads, with the median length ranging from 10 to 100 kb [141,142], but are also capable of achieving read lengths up to hundreds and thousands of kbs [143], so that one read could contain >20 concatenated transgene copies. ONT was recently employed to sequence transgenic mouse lines with interesting integration patterns [56,88,144], signifying a start of a new era in mouse genome analysis. We have used both methods to study concatenation of barcoded library in the embryos [40]; unpublished data) and discovered plenty of complex internal concatemer structures. 

It should be noted that SMS technologies suffer from two critical limitations that stall their utility for transgene concatenation analysis. First of all, these methods are still very expensive for analyzing transgene inserts. Even large concatemers spanning 0.1–1 Mb in size correspond to only about 0.02% of the mouse diploid genome, which will make up a small fraction of the total sequencing data. The results of a typical ONT/PacBio run usually constitute 1–5x mouse genome coverage [56,88,144] and it could be difficult to assemble the whole concatemer from these data. We have a performed transgene sequences experiment for concatemers with high copy numbers (>100). The ONT MiniON device produced about 8 Gb, which contained around 0.9 Mb of transgene sequences, equal to 0.02%, and contained about 50 unique copies (50%) (unpublished data). In an alternative PacBio experiment with another transgenic line, we obtained 16.1 Gb, of which 1.16 Mb (0.07%) corresponded to the transgene sequence and contained around 90 unique copies (80%) [40]. 

For this reason, SMS methods are frequently paired with target enrichment to increase the transgene read percentage. Target DNA capture with sequence-specific biotinylated probes can provide extremely high enrichment ratios (10^3^–10^5^) at the cost of simplicity [145]. Today, most common techniques for ONT and PacBio involve cleaving target transgene sites in dephosphorylated genomic DNA samples with CRISPR/Cas9 nuclease and ligating adapter for sequencing to the phosphorylated ends [136,137,138]. This is a powerful approach that could increase transgene read counts 50–60× [138,146] with up to 1600× in some cases [144], but it is not applicable for concatemer studies, because fragmenting of the internal structure is undesirable. With this in mind, we could appreciate DNA barcodes as unique Cas9 target sites that could be used for transgene enrichment. In theory, concatemer structure loss could also be avoided by mapping integration sites first [147], using these flanking sites as Cas9 targets, and subsequent DNA fragmentation, consequently decreasing the size of the enriched concatemer fragment to affordable lengths. 

The second SMS limitation is the high error rate. Current routine applications achieve around 90–95% accuracy for both technologies [148,149], which might be enough for general transgene mapping, but is absolutely deadly for the short barcode identification. In the aforementioned experiments, we observed an 83% accuracy for ONT and 92% for PacBio reads; however, it did not help to reliably identify barcodes from long reads and we had to rely on the NGS data for validation. In addition, current error rates obstruct interpretation of nucleotide sequences at transgene-transgene junctions, limiting the value of sequencing data. Again, simply increasing the sequencing depth by upscaling or target enrichment could be useful as both technologies profit from consensus from multiple reads and reach >99% accuracy [150,151,152], but it could be costly. 

Furthermore, new evidence warns that palindromes might represent a challenge for ONT sequencers, because the secondary DNA structure affects strand transfer dynamics through the pore [153]. This is arguably a minor drawback but has to be addressed to study palindromic junctions in detail. How the PacBio approach treats palindromic regions has not been systematically analyzed yet. In our own PacBio data, we did not see a palindrome-associated drop of quality during concatemer sequencing [40]. 

In conclusion, SMS methods offer unprecedented opportunities to study internal concatemer structure when combined with DNA barcoding but require improvements to be useful in the high-throughput analysis of transgenic inserts.

## 7. Conclusions and Future Direction

Repair of double-strand breaks during zygotic division is very poorly characterized [154,155]. In general, it is considered that the early embryo has high baseline HR rates, because the cell cycle is shifted towards S/G2 phases [156].

Many known integrated DNA reporters could be combined with pronuclear injection to study the dynamics of DNA repair during zygotic division. For example, barcoded transposons [157] or fluorescence-based reporters [158] are used to calculates outcomes of DSB repair (HR, NHEJ, MMEJ) in cells and could be adapted for generation of transgenic mice for such experiments. Alternatively, droplet digital PCR-based assays could provide a convenient tool to study Cas9 indel signatures at any genomic sites in the embryo [159,160]. These reporters could also be used in ES cells or other cell culture types to directly compare repair efficiencies at specific sites with the embryo.

Concatenation assays represent a convenient alternative to integrated reporters and could provide plenty of data for the fundamental studies of DNA repair. The advantages of the pronuclear microinjection over cell culture experiments include an opportunity to choose the timing of zygote development. For example, injections in pre-replicative early pronucleus, late G2-stage pronucleus, or in the two-cell stage embryos show distinct outcomes in gene-targeting experiments [156,161]. A barcoded concatemer system would be a convenient way to evaluate HR/NHEJ activities at these stages.

Finally, barcoded DNA injections could be combined with powerful methods, such as auxin-dependent protein degradation [162], co-injections of the DNA repair factors (Rad51, Rad18, CtIP, Ku) [155,163,164,165], or small molecule inhibitors [166], to dynamically alter the DNA damage balance and discover factors responsible for transgene end recombination.

From the angle of biotechnology, understanding concatenation mechanisms is highly desired to control the outcome of the experiments both in manufacturing cell lines and in animal transgenesis. For commercial cell lines, rearranged concatenated transgene integrants often result in genetically unstable loci [14], potentially prone to RIGS. Identifying epigenetic factors responsible for RIGS will help to achieve reliable transgene expression through chemical inhibition, as was shown in the extrachromosomal circles model with HDAC inhibitors [6]. Moreover, preventing concatenation altogether by limiting end recombination with biotin [84] or inhibiting HR [167] are promising strategies to avoid rearrangements in the gene-targeting experiments. Lastly, one may even fantasize that better control over concatenation will help to assemble and integrate megabase-sized transgenes directly in an injected zygote in a Gibson cloning style [37,168], possibly using long single-stranded DNA fragments [169,170] that are preferred substrates for HR and less susceptible to random ligation.

Concatenation studies is a nice example of resurrection of the suspended mystery with new genomic technologies, which will undoubtedly bring many new discoveries about DNA recombination in the nucleus.
